# Electroanatomically estimated length of slow pathway in atrioventricular nodal reentrant tachycardia

**DOI:** 10.1007/s00380-013-0424-0

**Published:** 2013-10-13

**Authors:** Tadanobu Irie, Yoshiaki Kaneko, Tadashi Nakajima, Masaki Ota, Takafumi Iijima, Mio Tamura, Takashi Iizuka, Shuntaro Tamura, Akihiro Saito, Masahiko Kurabayashi

**Affiliations:** Department of Medicine and Biological Science, Gunma University Graduate School of Medicine, 3-39-22 Showa-machi, Maebashi, Gunma 371-8511 Japan

**Keywords:** Atrioventricular nodal reentrant tachycardia, Slow pathway, Koch’s triangle, Atrioventricular node, Radiofrequency ablation, Electroanatomic mapping

## Abstract

The length of the slow pathway (SP-L) in atrioventricular (AV) nodal reentrant tachycardia (NRT) has never been measured clinically. We studied the relationship among (a) SP-L, i.e., the distance between the most proximal His bundle (H) recording and the most posterior site of radiofrequency (RF) delivery associated with a junctional rhythm, (b) the length of Koch’s triangle (Koch-L), (c) the conduction time over the slow pathway (SP-T), measured by the AH interval during AVNRT at baseline, and (d) the distance between H and the site of successful ablation (SucABL-L) in 26 women and 20 men (mean age 64.6 ± 11.6 years), using a stepwise approach and an electroanatomic mapping system (EAMS). SP-L (15.0 ± 5.8 mm) was correlated with Koch-L (18.6 ± 5.6 mm; *R* ^2^ = 0.1665, *P* < 0.005), SP-T (415 ± 100 ms; *R* ^2^ = 0.3425, *P* = 0.036), and SucABL-L (11.6 ± 4.7 mm; *R* ^2^ = 0.5243, *P* < 0.0001). The site of successful ablation was located within 10 mm of the posterior end of the SP in 38 patients (82.6 %). EAMS-guided RF ablation, using a stepwise approach, revealed individual variations in SP-L related to the size of Koch’s triangle and AH interval during AVNRT. Since the site of successful ablation was also correlated with SP-L and was usually located near the posterior end of the SP, ablating anteriorly, away from the posterior end, is not a prerequisite for the success of ablation procedures.

## Introduction

Ablation of the slow pathway (SP) is a curative treatment of atrioventricular (AV) nodal reentrant tachycardia (NRT) [1–6]. Lesions are usually created from the right side of the septum in the inferior or mid segment of the triangle of Koch [1–6], although the precise site of successful ablation is found over a wide midseptal or posteroseptal region [1, 5–7]. Some clinical investigators have suggested that the highly variable sizes and shapes of Koch’s triangle [8–10], and size and morphology of the coronary sinus (CS) ostium [11, 12], contribute to the variability in the location of successful ablation sites among patients. In particular, there are anatomic variations in the right posterior extension of the AV node, which act as SP during ongoing AVNRT [13], although neither their location relative to Koch’s triangle or the CS ostium nor the length of the SP (SP-L) have been evaluated clinically. The identification of the precise anatomic location of SP is likely to optimize the safety of its ablation. Moreover, the methodological determinants of this individual variability in previous studies might be explained, at least in part, by the limited accuracy of fluoroscopy in identifying anatomic landmarks and the optimal ablation site [1, 5–7, 9–12, 14–16]. An electroanatomic mapping system (EAMS) might precisely track the tip of the mapping catheter and accurately measure the distance between two points in a three-dimensional space [17, 18]. This study examined the relationships among SP-L, the dimensions of Koch’s triangle, the successful ablation site, and the conduction time over SP, in search of predictors of interindividual variations at the site of successful SP ablation in AVNRT, using an EAMS.

## Patients and methods

### Study population

We enrolled 26 women and 20 men, 64.6 ± 11.6 years of age, who presented with slow–fast AVNRT and underwent electrophysiologic studies and SP ablation. A slow–slow form of AVNRT was also observed in one patient, and no patient presented with the fast–slow form of AVNRT. The study complied with the guidelines of the declaration of Helsinki and was approved by the institutional review board of Gunma University Hospital. All patients granted their written informed consent to participate in this study.

### Electrophysiologic studies

The patients underwent baseline electrophysiologic studies in the fasting, nonsedated state, after discontinuation of all antiarrhythmic drugs for ≥5 half-lives. Four multipolar electrode catheters were placed in the high right atrium, His-bundle area, the right ventricle, and CS, for intracardiac recordings and for pacing. AV conduction during incremental atrial and ventricular pacing and induction of AVNRT by extrastimulation were studied in detail. Intravenous isoproterenol was infused in doses between 1 and 4 μg/min to facilitate the induction of AVNRT when only pacing maneuvers were unsuccessful. AVNRT was diagnosed according to previously published criteria [19].

### Definitions of anatomic landmarks

**Fig. 1:**
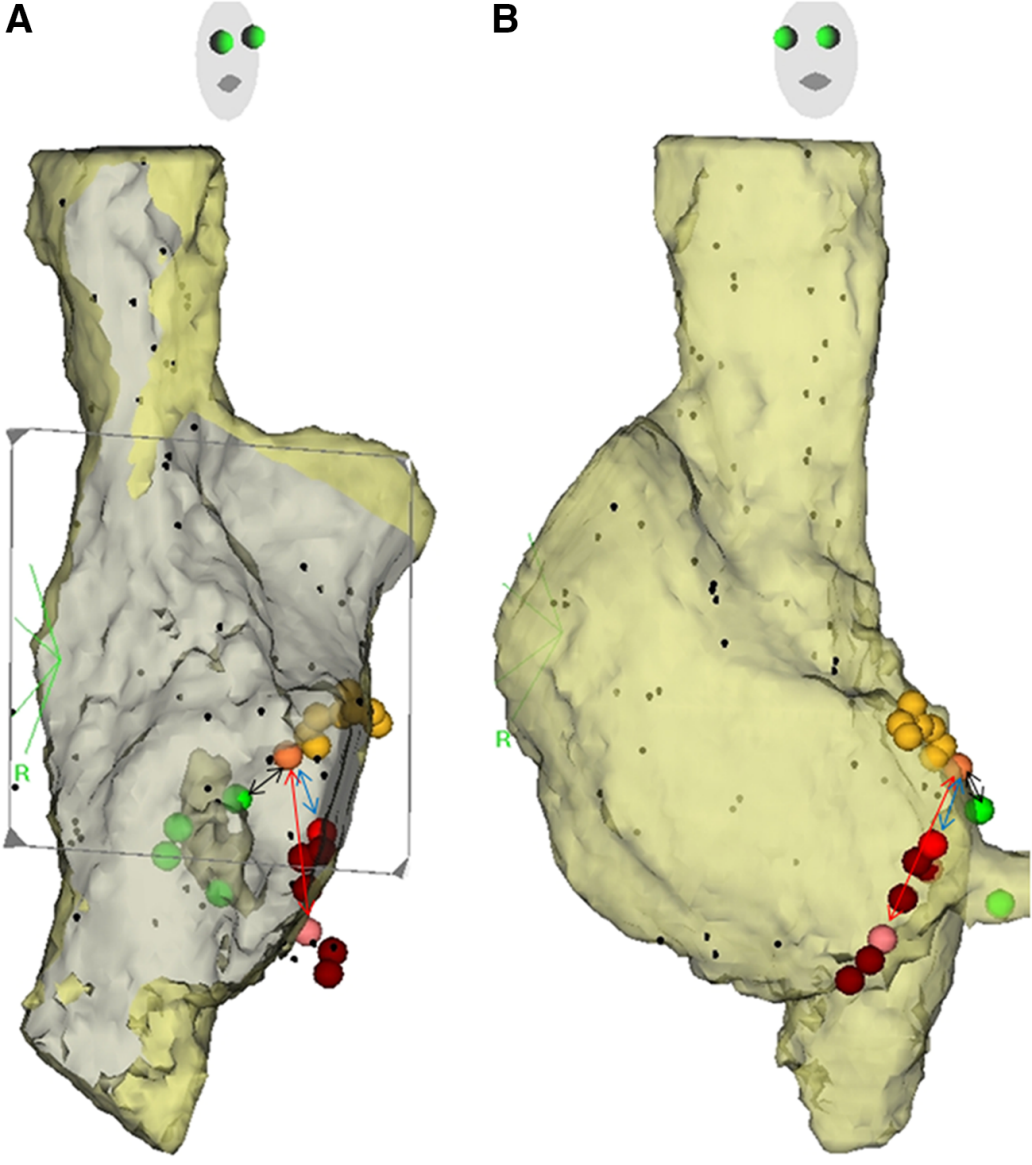
Right atrial endocardial electroanatomic mapping superimposed on computed tomographic images in the right (**a**) and left (**b**) oblique projections. The *orange and yellow tags* show the site of the most proximal His-bundle electrogram and all other sites of His electrogram recordings, respectively; the *pink, red, and brown tags* show the most posterior site associated with junctional rhythm during radiofrequency (RF) delivery, the site of successful ablation, and all other sites of RF delivery, respectively. The *green tags* show the superior and inferior margins of the CS ostium. The *bidirectional black, red, and blue arrows* show the length of Koch’s triangle, length of slow pathway, and distance between His bundle and the site of successful ablation, respectively (color figure online)

At baseline, we mapped the entire right atrium during sinus rhythm, using a CARTO X-P EAMS and a 7F deflectable, nonirrigated Navistar catheter with a 4 mm-tip (Biosense Webster, Diamond Bar, CA, USA) and recorded the location of the most proximal and distal His-bundle electrograms (H), between the proximal right bundle branch and the superior and inferior margin of the CS ostium, defined as the junction of the CS with the right atrium, and confirmed by fluoroscopy in the left and right anterior oblique views (Fig. 1) [20].

The definitions used in previous anatomic studies of the dimensions of Koch’s triangle have been inconsistent [8, 21, 22]. Therefore, as in previous clinical studies [9, 10], we measured the distance between the earliest H recording and the superior margin of the CS ostium (Fig. 1) as the length of Koch’s triangle (Koch-L) [23].

### Electrophysiologic measurements

The baseline conduction time through the SP in the nonsedated, drug-free state, without infusion of isoproterenol, was defined as (a) in patients presenting with discontinuous AV conduction curves, the first “jump” in A2–H2 interval during premature atrial stimulation (SP-T1) following trains of six fixed paced S1–S1 cycles at the shortest cycle length associated with 1:1 A1–H1 conduction, and (b) the AH interval (i.e., the time between the onset of the first rapid atrial deflection and the His bundle deflection) during AVNRT (SP-T2) [24]. The measurements during infusion of isoproterenol were not included in the analysis of conduction time over the SP. Since the entrance to the SP is generally located near the CS ostium, the AH interval was measured between the earliest H recording and the atrial electrogram recorded from the proximal bipole of the CS catheter located near the CS ostium, providing the most accurate SP conduction time [24, 25].

### Slow pathway ablation and length of the slow pathway

To locate the posterior end of the SP and measure its length, we used a combined anatomic and electrogram-guided method [6]. Radiofrequency (RF) energy with a maximum output of 30–50 W, a maximum temperature of 50–55 °C, and a maximum duration of 30–40 s was first delivered to the inferoposterior aspect of the interatrial septum near the inferior CS ostium, where a <1.0 atrial/ventricular deflection ratio was recorded from the distal tip of the ablation catheter. An SP potential was not used as a guide. If an accelerated junctional rhythm was not observed during the ablation attempt, RF was delivered at a recording site associated with a slightly greater atrial/ventricular ratio at the same level between the His bundle and the CS ostium, or 1–2 mm higher and more anteriorly along the tricuspid annulus until the development of a junctional rhythm, with a view to reach the most proximal (atrial) insertion of the SP. In addition, energy application was terminated as soon as any evidence of AV or ventriculoatrial block was detected. After each RF application associated with the development of an accelerated junctional rhythm, programmed stimulation was performed to reassess the quality of anterograde AV nodal conduction and confirm the noninducibility of AVNRT. If AVNRT remained inducible, RF deliveries were repeated, using a similar stepwise approach, until successful ablation, defined as <2 inducible reentrant cycles before and during the infusion of isoproterenol [16, 26].

Based on previous studies, which found that the course of SP is posterior and inferior along the tricuspid annulus [13], and that an accelerated junctional rhythm occurring during delivery of RF energy results from thermally mediated enhanced automaticity of either AV nodal or perinodal tissue with underlying pacemaker activity [27, 28], we defined the most posterior site of RF delivery associated with an accelerated junctional rhythm as the end of the SP, and measured the distance between the earliest H recording and the end of the SP (Fig. 1) as the estimated length of the SP (SP-L). Accelerated junctional rhythm was defined as any sequence of premature depolarizations propagating from the AV junctional region in a centrifugal direction. We analyzed the characteristics of the rhythm during RF application at the posterior end of the SP and at the site of successful ablation, including the mean number of junctional cycles and the total number of cycles/30 s, the time from the initiation of RF energy delivery to the onset of accelerated junctional rhythm, the total duration of junctional rhythm, and the temperature and power at the onset of the rhythm [29, 30]. We also (a) measured the distance between the earliest H recording and the site of last RF application (SucABL-L) after which successful ablation (as defined earlier) was confirmed (Fig. 1), and (b) classified the septal tricuspid annulus between the anterior CS ostium and the earliest H as inferior, medial, or superior [5, 7].

### Statistical analysis

The measurements are expressed as mean values ± standard deviation (SD). The correlations between pairs of measurements, including Koch-L, SP-L, SucABL-L, SP-T1, and SP-T2 were examined by linear regression analysis. *P* < 0.05 was considered statistically significant.

## Results

### Electrophysiologic measurements

The ablation procedure was successful in all patients, using a mean of 7.7 ± 6.8 RF applications (range 2–39). An atrial/ventricular ratio at the site of successful ablation was significantly greater than that at the posterior end of the SP (0.29 ± 0.27 vs 0.22 ± 0.22, *P* = 0.0358). Table 1 shows the electrophysiologic measurements before and after SP ablation. The mean tachycardia cycle length was 415 ± 100 ms in 13 patients whose tachycardia was inducible without isoproterenol, and 341 ± 45 ms in 33 patients whose tachycardia was inducible with infusion of isoproterenol. SP conduction was eliminated in five patients (11 %). Anterograde conduction over the fast pathway remained intact in all patients, although its effective refractory period was significantly shorter after compared with before the ablation procedure (Table 1).

**Table 1:** Electrophysiologic measurements before and after slow pathway ablation

	Before	After	*P*
AH interval during sinus rhythm (ms)	80 ± 25	82 ± 21	ns
Slowest atrial pacing rate associated with AH block (ppm)	165 ± 30	165 ± 28	ns
Effective refractory period (ms)			
Fast pathway	356 ± 83	334 ± 71	0.045
Slow pathway	274 ± 59	258 ± 71	ns
AH interval at 1st jump without isoproterenol (SP-T1) (ms)	300 ± 107	NA	–
Tachycardia cycle length (ms)			
Without isoproterenol (SP-T2) (*n* = 13)	415 ± 100	NA	–
During isoproterenol infusion (*n* = 33)	341 ± 45	NA	–
AH interval during tachycardia (ms)			
Without isoproterenol (*n* = 13)	345 ± 82	NA	–
During isoproterenol infusion (*n* = 33)	297 ± 42	NA	–

Values are mean ± SD

*NA* not applicable

### Site of successful ablation, size of Koch’s triangle, and length of slow pathway

**Fig. 2:**
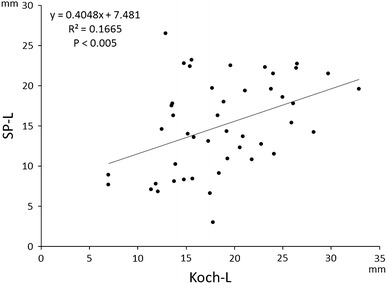
Correlation between the length of slow pathway (SP-L) and the size of Koch’s triangle (Koch-L)

The mean SP-L was 15.0 ± 5.8 mm (range 3.0–26.5 mm) and mean Koch-L 18.6 ± 5.6 mm (range 7.0–32.9 mm). There was a moderate correlation between SP-L and Koch-L (*R* ^2^ = 0.1665, *P* < 0.005; Fig. 2). In 37 patients (80 %), SP-L was shorter than Koch-L, indicating that SP was anterior to the superior margin of the CS ostium, while in nine patients (20 %) SP-L was longer than Koch-L, indicating that it was posterior to the superior margin of the CS ostium. The characteristics of the junctional rhythm during RF application at the posterior end of the SP, except for the number of junctional cycles/30 s, were similar to those observed at the site of successful ablation (Table 2).

**Table 2:** Characteristics of junctional rhythm during radiofrequency application at the posterior end of slow pathway and the site of successful ablation

	Posterior end of slow pathway	Site of successful ablation	*P*
No. of junctional cycles/30 s	12.6 ± 10.4	17.5 ± 15.2	0.046
Interval between initiation of RF delivery and onset of accelerated junctional rhythm (s)	14.0 ± 6.1	14.0 ± 6.8	ns
Overall duration of junctional rhythm (s)	7.0 ± 5.8	10.0 ± 8.8	ns
Mean length of junctional cycles (ms)	500 ± 238	510 ± 198	ns
Temperature at onset of accelerated junctional rhythm (°C)	45.5 ± 4.5	45.9 ± 4.3	ns
Power at the onset of accelerated junctional rhythm (W)	22.4 ± 10.8	23.1 ± 11.3	ns

Values are mean ± SD

The mean SucABL-L was 11.6 ± 4.7 mm (range 3.0–20.3 mm). The SucABL-L was ≤5.0 mm in 3 patients (7 %), between 6.0 and 10.0 mm in 17 (37 %), 11.0 and 15.0 mm in 13 (28 %), 16.0 and 20.0 mm in 12 (26 %), and >21.0 mm in 1 (2 %) patient. SucABL-L and Koch-L were correlated (*R* ^2^ = 0.1183, *P* = 0.019). In 42 patients (91 %), the SucABL-L was shorter than the Koch-L, indicating that the site of successful ablation was anterior to the superior margin of the CS ostium, while in four patients (9 %) the SucABL-L was longer than Koch-L, indicating that is was posterior to the superior margin of the CS ostium. Furthermore, the site of successful ablation was in the inferior region in 15 (33 %), medial region in 29 (63 %), and superior region in 2 (4 %) patients. These observations suggest that although SP-L and SucABL-L were both correlated with Koch-L, neither the posterior end of the SP nor the site of successful ablation were confined to the anterior region of the inferior CS ostium margin, and that both varied among individual patients.

### Conduction across and length of slow pathway, and site of successful ablation

**Fig. 3:**
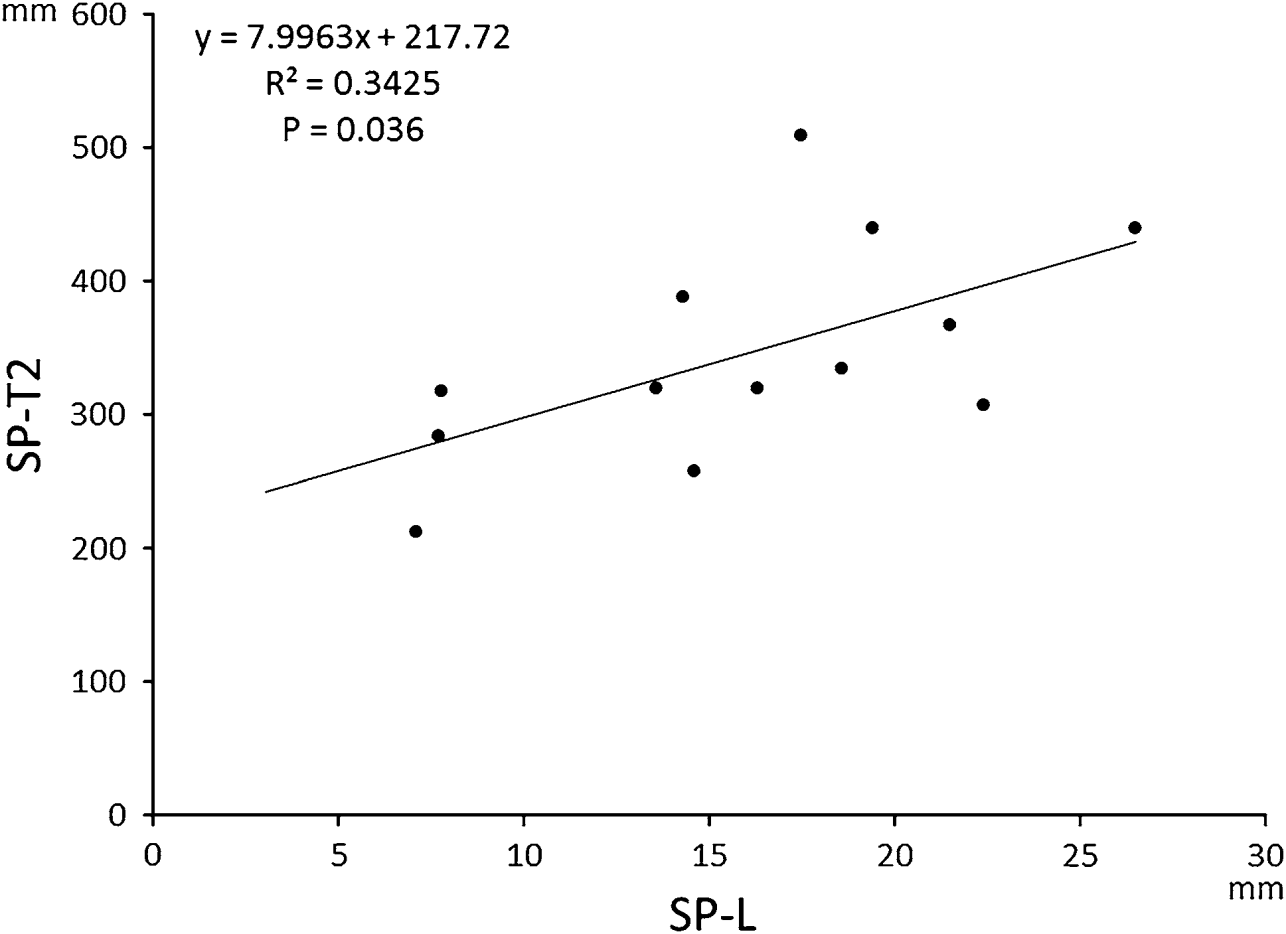
Correlation between the length of slow pathway (SP-L) and the AH interval during the tachycardia (SP-T2)

The cycle length of regular pacing preceding atrial extrastimulation was 600 ms in 42 patients, 500 ms in 2, 400 ms in 1, and 750 ms in 1 patient. We found correlations neither between SP-T1 and SP-L nor between SP-T1 and SucABL-L (data not shown). There was, however, a correlation between SP-T2 and SP-L (*R* ^2^ = 0.3425; *P* = 0.036; Fig. 3), although there was no correlation between SP-T2 and SucABL-L (data not shown). The mean conduction velocity over SP, estimated by dividing SP-T2 by SP-L, was 4.6 ± 1.5 cm/s (range 2.7–7.3 cm/s).

### Relationship between length of slow pathway and distance between His bundle and site of successful ablation

**Fig. 4:**
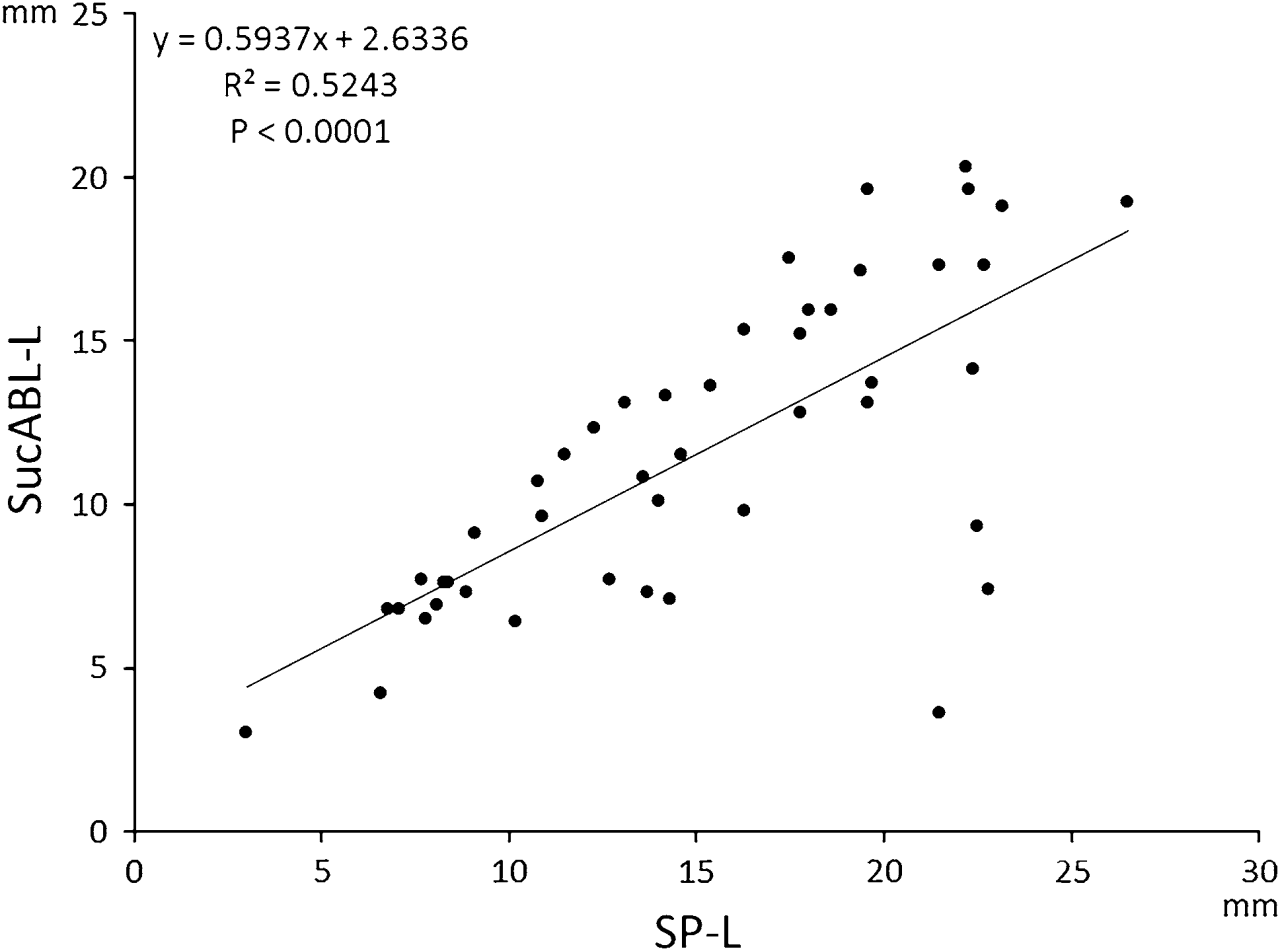
Correlation between the length of slow pathway (SP-L) and the distance between His-bundle electrogram and the site of successful ablation (SucABL-L)

SP-L and SucABL-L were strongly correlated (*R* ^2^ = 0.5243, *P* < 0.001; Fig. 4). The mean distance between the posterior end of the SP and the site of successful ablation was 6.2 ± 4.2 mm, and was <5 mm in 19 (41.3 %), 5–10 mm in 19 (41.3 %), and >10 mm in 8 (17.4 %) patients. Therefore, the site of successful ablation was located within 10 mm of the posterior end of the SP in 38 patients (82.6 %), suggesting that the site of successful ablation was (a) related to the SP-L, (b) was located near the posterior end of the SP in most patients, and (c) was located proximally, away from the posterior end of the SP in a minority of patients.

## Discussion

The main findings of the present study were: (1) SP-L was positively correlated with Koch-L and with the conduction time over SP during tachycardia; (2) SP was strictly anterior to the CS ostium in most patients, and extended posteriorly beyond the superior margin of CS ostium in only few patients; and (3) the site of successful ablation was (a) associated with SP-L, (b) located near the posterior end of the SP in most patients, and (c) located away from and proximal to the posterior end of the SP in a minority of patients.

### Measurement of length of the slow pathway

To our knowledge, this is the first study in which the SP-L was calipered during RF ablation of AVNRT. Since it was successfully ablated strictly from the right side, without any RF delivered from inside the CS ostium, SP seems anatomically consistent with a right posterior extension, identified as a discrete posterior continuation of the compact AV node [14]. Furthermore, the fewer junctional cycles observed during RF application at the posterior end of the SP compared with the site of successful ablation (Table 2) might reflect differences in thermally mediated enhanced automaticity between the perinodal tissue and the AV nodal tissue near the compact AV node. Thus we believe that, when using a stepwise approach along the tricuspid annulus, the posterior end of the right posterior extension could be detected by triggering the junctional rhythm as a marker of AV nodal tissue ablation [27–30]. SP-L, which in the present study was measured precisely between the most proximal H and the proximal end of the SP, might have reflected the actual conduction across the SP during tachycardia. Although whether SP-L is similar in patients with versus without AVNRT needs to be verified, the individual variations in SP-L observed in the present study may reflect the expected anatomic variations of a normal AV node.

### Relationship between size of Koch’s triangle and slow pathway

As observed in previous anatomic and clinical investigations [9, 13], the dimensions of Koch’s triangle varied considerably among our patients, and were similar to the mean 17 ± 3 mm, which was measured post mortem and during surgery [21], suggesting that we studied a typical population. In contrast to previous studies, which used two-dimensional fluoroscopy to measure Koch-L [9, 10, 16], our calipered measurements by electroanatomic mapping were likely to reflect accurately its individual variations. It is noteworthy that our population included two patients who underwent successful ablation posterior to the superior margin of the CS ostium, whose Koch-L was <10 mm.

An accurate prediction of the anatomy of the AV node before ablation of AVNRT in each individual patient would be helpful, especially the location of the right posterior extension in Koch’s triangle. Several anatomic determinants of successful SP ablation have been identified [7, 9, 14–16]; however, the precise location of the SP in Koch’s triangle has not been reported in a clinical setting. In the present study, SP-L was correlated with Koch-L, indicating that the latter was a predictor of SP-L. However, the location of the posterior end of the SP was not confined to the anterior region of the inferior CS ostium margin, suggesting that such an anatomic landmark cannot simply be used to predict the location of the posterior end of the SP. Inoue and Becker [13] found that, in most cases, the AV nodal bundle axis reaches the level of the anterior margin of the CS ostium, and that in one-third of cases, the posterior bundle extends beyond the anterior margin of the CS ostium, which is consistent with our observations.

### Relationship between length of slow pathway and conduction time over slow pathway

In the present study, the AH interval during AVNRT without infusion of isoproterenol was positively correlated with SP-L. These findings suggest that anatomic factors, such as SP-L contribute to differences in the AH interval during tachycardia observed among patients, besides functional factors, such as cellular electrophysiologic characteristics and autonomic nervous activity. In contrast to these previous studies [24, 31, 32], the AH interval during tachycardia in our study was not correlated with the site of successful ablation. This is probably (a) because in some patients, the site of successful ablation was not consistent with the precise location of the posteroseptal atrionodal input (entrance) of SP [25], as described in detail later, and/or (b) a consequence of methodological differences in the localization of the successful ablation site. In addition, the AH interval at the first AH jump (SP-T1) was not indicative of the SP-L, probably because decremental conduction over SP at the time of jump was variable among patients. The calculated mean 4.6 ± 1.5 cm/s conduction velocity across SP without infusion of isoproterenol closely resembled the 6.9 ± 2.4 cm/s measured in the rabbit heart, using fluorescent imaging of AV nodal reentry [33].

### Relationship between length of slow pathway and site of successful ablation

Although one might hypothesize that our successful ablation site was in a more superior location than previously described [3, 9], we believe that it was nearly identical despite differences in (a) the calipering between fluoroscopic and EAMS guidance and (b) the starting point of the interval between the largest and the proximal His-bundle electrogram. Importantly, a positive linear correlation between SP-L and SucABL-L suggests that (a) SP-L may be one of the factors responsible for individual variations in the site of successful ablation, and (b) successful SP ablation was achieved by selectively targeting the posterior atrionodal input in most patients, as in the study of Markowitz et al. [34]. However, in a minority of patients, ablation was successful away from and anterior to the posterior end of the SP, following ineffective RF applications near its posterior end. This might have two distinct explanations. First, assuming that only part of SP, which was wider at its entrance than at other more superior sites [34], functioned as an essential component of the tachycardia circuit, a point ablation at its posterior end might have caused an incomplete lesion, allowing the persistence of AVNRT. Alternatively, in some patients, instead of being located along the tricuspid annulus near the inferior CS ostium, the SP might have been located between the CS ostium and the tricuspid annulus. In such cases, a linear ablation across the wide entrance of SP might eliminate the tachycardia [16]. Second, SP might run deep in the endocardium, not readily reached by RF applications from the right atrium. We believe that such an SP might be a variant of a right posterior instead of a left inferior extension since, in contrast to earlier studies [2, 3, 5, 15], none of our patients needed the delivery of RF from the left side or from inside the CS ostium to ablate a left inferior extension [1, 6, 7, 35, 36].

### Clinical implications

Albeit low, the risk of AV block complicating ablation of the SP is not negligible [1–7]. One of the possible causes of this complication might be that the same conventional techniques, whether anatomically or electrogram-guided, are systematically implemented in each case, irrespective of the individual SP-L. The present study, however, identified interindividual variations in SP-L, which were correlated with Koch-L, AH interval during AVNRT, and the site of successful ablation. This new information suggests that a patient-based strategy might be more appropriate. For example, the observation of a long Koch-L or a long AH interval during AVNRT would encourage a posterior ablation, away from the His-bundle, whereas the finding of a short Koch-L or AH interval would be indicative of a heightened risk of AV nodal injury. In addition, our observations suggest that ablating anteriorly, away from the posterior end of the SP, was not a prerequisite for successful ablation procedures. This strategy, therefore, might be a means of lowering to a minimum the risk of AV block as well as the number of unnecessary RF applications.

### Study limitations

A first limitation of our study is the definition of the posterior end of the SP. There is currently no evidence of an anatomic correlation between the sites of induction of junctional rhythm and the most atrial end of the SP. In addition, the AV nodal or perinodal tissue during RF delivery might be heated by both resistive and conductive heat. When the SP is in a deep endocardial layer, the heating effect might not reach its posterior end. Furthermore, no junctional activity might occur during RF application at the site of successful ablation [37]. In these unusual circumstances, the most posterior site associated with the development of junctional rhythm during RF application might not be the posterior end of the SP. Second, the AH interval during tachycardia was measured in only one-third of the population whose tachycardia was inducible at baseline. Third, although the actual SP-T was between the earliest His electrogram and the atrial electrogram at the sites of successful ablation, we did not map that atrial electrogram during the tachycardia. Fourth, we did not evaluate the output, duration of RF energy, and tissue temperature on the development of junctional rhythm during RF application.

## Conclusions

Ablation guided by an EAMS, using our safe and successful stepwise approach, detected individual variations in SP-L, which were correlated with Koch-L and SP-T2. In most patients, since SucABL-L was also correlated with SP-L and the site of successful ablation was located near the posterior end of the SP, ablating anteriorly, away from the posterior end of the SP, does seem to be a prerequisite for procedural success.
